# A Deep Learning Approach for the Morphological Recognition of Reactive Lymphocytes in Patients with COVID-19 Infection

**DOI:** 10.3390/bioengineering9050229

**Published:** 2022-05-23

**Authors:** José Rodellar, Kevin Barrera, Santiago Alférez, Laura Boldú, Javier Laguna, Angel Molina, Anna Merino

**Affiliations:** 1 Department of Mathematics, Barcelona Est Engineering School, Universitat Politècnica de Catalunya, 08019 Barcelona, Spain; kevin.barrera@upc.edu; 2 School of Engineering, Science and Technology, Universidad del Rosario, Bogotá 111711, Colombia; edwin.alferez@urosario.edu.co; 3 Biomedical Diagnostic Center, Core Laboratory, Department of Biochemistry and Molecular Genetics, Hospital Clinic de Barcelona, 08036 Barcelona, Spain; boldu.laura@gmail.com (L.B.); jlaguna@clinic.cat (J.L.); amolinab@clinic.cat (A.M.); amerino@clinic.cat (A.M.)

**Keywords:** deep learning, convolutional neural networks, COVID-19, blood cell images, cell morphology, reactive lymphocytes, diagnosis, prognosis

## Abstract

Laboratory medicine plays a fundamental role in the detection, diagnosis and management of COVID-19 infection. Recent observations of the morphology of cells circulating in blood found the presence of particular reactive lymphocytes (COVID-19 RL) in some of the infected patients and demonstrated that it was an indicator of a better prognosis of the disease. Visual morphological analysis is time consuming, requires smear review by expert clinical pathologists, and is prone to subjectivity. This paper presents a convolutional neural network system designed for automatic recognition of COVID-19 RL. It is based on the Xception71 structure and is trained using images of blood cells from real infected patients. An experimental study is carried out with a group of 92 individuals. The input for the system is a set of images selected by the clinical pathologist from the blood smear of a patient. The output is the prediction whether the patient belongs to the group associated with better prognosis of the disease. A threshold is obtained for the classification system to predict that the smear belongs to this group. With this threshold, the experimental test shows excellent performance metrics: 98.3% sensitivity and precision, 97.1% specificity, and 97.8% accuracy. The system does not require costly calculations and can potentially be integrated into clinical practice to assist clinical pathologists in a more objective smear review for early prognosis.

## 1. Introduction

Peripheral blood (PB) carries several cell types suspended in plasma, all essential for immunity and life: erythrocytes, leukocytes, and platelets. Leukocytes include neutrophils, eosinophils, basophils, lymphocytes, and monocytes. Fortunately, circulating blood is easily accessible and visual cell inspection is very relevant in the working flows of clinical laboratories. Over the years, clinical pathologists, through visual inspection using the optical microscope, identify qualitative morphological traits to characterize the different leukocytes, as well as the types of abnormal cells, whose presence in blood is evidence of serious diseases such as leukemia and lymphoma, among others [1]. A drawback of visual morphological analysis is that it is time consuming and requires expert pathologists to review smears objectively and reliably, and is prone to inter-observer variability. Most morphological descriptions are given in qualitative (linguistic) terms and there is a lack of quantitative measures.

Image analysis, quantitative morphological features, and machine learning approaches have been the main technological tools adopted in the last decade to overcome these drawbacks [2]. The late explosion of deep learning has shifted the focus to new classification models that use convolutional neural networks (CNN) [3]. Unlike previous machine learning methods, automatic blood cell classification does not explicitly depend on complex segmentation of cell regions of interest and further feature selection.

Lymphocytes are the second most abundant among white blood cells and are essential for the adaptive immune system. From a functional point of view, lymphocytes can be divided into different types, mainly B and T. The function of B lymphocytes is related to the synthesis of antibodies that are responsible for humoral immunity. After being exposed to antigenic stimuli, they transform into B lymphocytes with immune memory. T lymphocytes represent 70% of all lymphocytes circulating in blood; they are responsible for the cell-mediated immunity and there are different subtypes. Reactive lymphocytes are T cells that exhibit morphological changes produced as a result of antigen stimulation, generally in response to viral infections.

COVID-19 is an infectious disease caused by the SARS-CoV-2 virus that has expanded in all continents. Laboratory medicine plays an essential role in its early detection, diagnosis, prognosis, and management [4]. Among hematology laboratory parameters, low lymphocyte counts are common, although with some variability [4,5]. Recent observations of blood cell morphology found the presence of reactive lymphocytes (RL) in some of the patients infected with COVID-19. They morphologically mimic RL found in other infections [6], but some of them show subtle morphological differences, such as a more basophilic cytoplasm and the occasional presence of small cytoplasmic vacuoles [7,8,9]. For the sake of clarity, in this paper, these lymphoid cells are called COVID-19 RL, and the reactive lymphocytes seen in other infections (viral, some bacterial, and protozoal infections) are referred to “Classic RL”.

In [10], a first model for the automatic recognition of COVID-19 RL was presented, suggesting that these lymphocytes could be detected by computerized approaches. Training and testing were performed on sets of cell images without considering individual patients. From a clinical point of view, these approaches would be really useful if, given an infected patient, a model could provide prognostic prediction based on analysis of the entire blood smear.

The objective of this work is to develop a new CNN-based model for the automatic recognition of COVID-19 RL in blood and perform an experimental evaluation with a set of blood smears from infected patients to conclude the ability of the system as a support tool for an early prognosis prediction of the disease.

Any patient presenting to the hospital with symptoms is subject to a screening blood test. Most of these patients present with quantitative alterations in the white blood cell count, which activates the visual morphological inspection protocol of the blood smear. Since the pandemic situation started, the need of the clinicians to know in advance biological data related to serious illness has motivated the identification of new biomarkers related to the prognosis of the infection. In this context, it is known that if the neutrophil count is predominant, this is related to a worse prognosis. Furthermore, previous publications showed that the presence of specific CD4 and CD8 T lymphocytes in COVID-19 infection is associated with less severe disease [11]. In addition, the study in [10] reported that if the number of lymphocytes is normal but there are some COVID-19 reactive lymphocytes, then the evolution of the patient has a better prognosis, suggesting a higher production of virus-specific T cells and a more intense response against the virus. Consequently, the laboratory findings from the inspection of the blood smear guide the clinical pathologist as to the status of the patient’s immune response to infection. COVID-19 is a new challenge and it may be relevant to have a new complementary morphological biomarker and a computerized aid to identify it.

From the point of view of the engineering approach, this work adopts an existing convolutional neural network architecture as a tool to obtain a set of quantitative descriptors from the images taken from patients’ smears. The Xception71 architecture is used through a comparative study among several other frameworks in the state of the art. At the end of the layered structure, the learned features are used by a fully connected perceptron with an output softmax function to obtain the probabilities of each predicted cell class. The full system is satisfactorily evaluated in a clinical setting, in which pathologists select a number of cells of the blood smear of a patient, which are passed through the classification model. The output is the prediction whether the patient belongs to the group associated with better disease prognosis.

This new contribution is clinically relevant. Since infection can progress from mild-moderate to severe disease, and even to critical illness characterized by acute distress respiratory syndrome apparition and multiorgan failure, it is urgent to identify prognosis factors that help predict the patient’s risk and control the disease. In this respect, the morphological analysis that detects the presence of COVID-19 RL in blood can be carried out at an early stage, as soon as the patient goes to a hospital. This presence does not have a direct impact on treatment, but, together with other clinical information, can help as a prognosis indicator.

### Related Work

An extensive research effort has been conducted within two years of COVID-19 emergence involving artificial intelligence (AI). Specifically, models based on deep learning have been proposed for the early detection, diagnosis, and prognosis of the disease, mainly using chest X-rays [12,13] and computed tomography (CT) scans of the chest [14,15].

X-rays are easier and more widely available, but CT gives three-dimensional imaging and is, therefore, preferable for evaluation and diagnosis of symptomatic patients. A multicenter project [16] reported an early study in which the use of a deep learning model was helpful in adding objectivity to the prediction of COVID-19 positive from chest CT images. A review on the state of the art of deep learning models using X-ray and CT techniques in COVID-19 was presented [17]. It was mainly focused on image databases, CNN architectures, performance metrics, and limitations of available approaches. A more recent comparative review on X-ray and CT scans using image processing along with deep learning was published [18], which includes more than 80 updated references.

The work presented in this paper is situated in a different scenario, which is that of laboratory medicine and more specifically in the branch dedicated to the cytological review of blood smears from patients.

The impact of artificial intelligence techniques in the hematological diagnosis has been increasing strongly [19,20] in the last decade. Here, we focus on machine learning and deep learning methods developed to automatically identify morphological patterns in cells circulating in the blood, which are associated with specific diseases, as this is the context of the presented work. The World Health Organization considers morphology, along with other complementary tests such as immunophenotype, cytogenetic, and molecular, essential for the integral diagnosis of hematological diseases. Advances in automated classification of digital microscopic cell images are important to complement visual morphological inspection by clinical pathologists, adding quantitative objectivity and consistency in the identification of complex patterns.

Some relevant examples of machine learning approaches focused on peripheral blood are the automated recognition of different types of leukocytes [21], the classification of abnormal lymphoid cells in different types of lymphoma [22], the differentiation between myeloblasts and lymphoblasts [23], as well as the classification of different types of acute myeloid leukemia (AML) [24] and acute lymphoid cell leukemia (ALL) [25,26].

Recently, CNN methodologies have been used to discriminate among the different normal leukocytes [27,28]. The recognition of acute leukemia with CNNs has been addressed mainly in two problems: (1) differentiate lymphoblasts and leukocytes with diverse cell morphology [29,30,31]; and (2) separate lymphoblast subtypes [32,33,34]. The work in [35] proposed a CNN model to distinguish neoplastic (leukemia) and non-neoplastic (infections) diseases, as well as to recognize the leukemia lineage. Automatic identification of hypogranulated neutrophils for the diagnosis of myelodysplastic syndromes has also been recently considered using CNN predictive models [36,37].

Malaria is a life-threatening disease caused by the Plasmodium parasite. The laboratory gold standard in the diagnosis of malaria is based on microscopic visualization of the parasite within infected erythrocytes in blood smear. Recently, different groups have addressed the automatic recognition of malaria-infected erythrocytes using machine and deep learning methods [38,39,40], including models that could be implemented on mobile devices [41,42]. The work in [43] proposed a new deep learning system capable of recognizing malaria-infected erythrocytes from normal erythrocytes and from erythrocytes with other types of inclusions. This approach helps reduce false positives, as other models tend to confuse other inclusions for the malaria parasite.

## 2. System Development

### 2.1. Overview

The purpose is to set up and train a classification system with the following inputs and outputs:

- Input: images of lymphocytes circulating in peripheral blood. They are acquired from a smear obtained from blood samples of patients.
- Output: their classification into Normal lymphocytes (NL), Classic RL, or COVID-19 RL.

**Figure 1:**
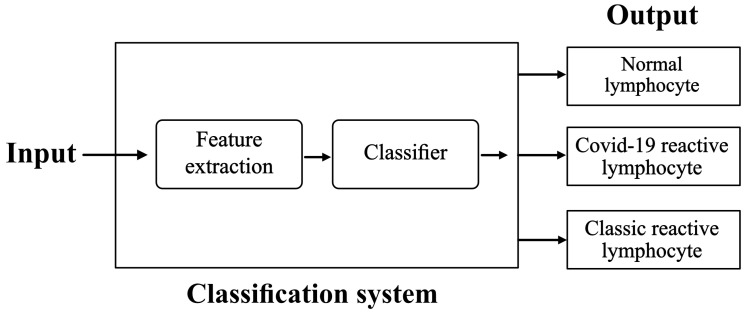
Classification system. The inputs are digital images of lymphocytes obtained from a fresh sample of the patient’s blood. The system includes a convolutional architecture for the extraction of quantitative features and a classifier to distinguish input cells as normal lymphocytes, COVID-19 RL, or Classic RL.

We propose the scheme illustrated in Figure 1.

**Figure 2:**
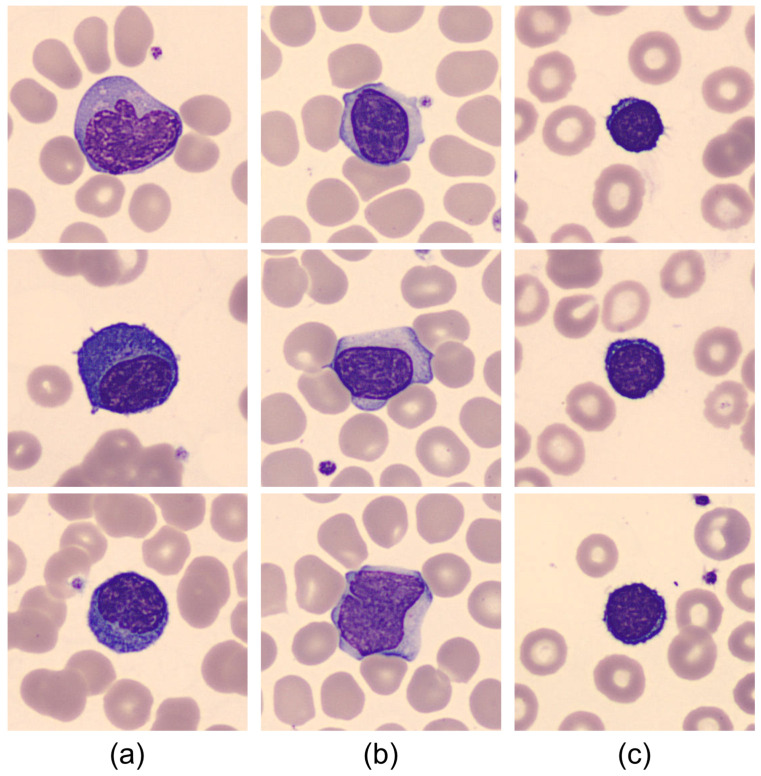
Examples of images of cells circulating in the blood of subjects involved in this study: (**a**) COVID-19 RL that have been found in some of the patients with COVID-19 infection; (**b**) Classic RL found in other viral infections; (**c**) Normal lymphocytes from healthy subjects. These images have been obtained from patients involved in this study.

There are subtle but distinctive morphological features between the three types of lymphocytes included in the study and illustrated with the example cell images shown in Figure 2. The usual way that clinical pathologists describe the cell morphology is in qualitative terms as follows. COVID-19 RL (a) show a deeper basophilic cytoplasm with occasional presence of small cytoplasmic vacuoles and an eccentric nucleus containing occasional nucleoli. Reactive lymphocytes (b) in classical infection are larger and show larger cytoplasm that is predominantly basophilic at the edges and adheres to neighboring red blood cells. Normal cells (c) are smaller in size and show a higher nucleus/cytoplasm ratio because the cytoplasm is scarce and the chromatin in the nucleus is mature. An experienced cytologist is able to differentiate between these kinds if cells based on these types of qualitative characteristics by visual inspection of the blood smear. However, this is prone to subjectivity and inter-observer variability and is time consuming. Furthermore, the morphological differences are very small in some cases, which requires great skill and experience.

In this work, we propose an automatic classification model based on convolutional neural networks (CNN). The conceptual paradigm is that the artificial system will be able to learn a set of quantitative features directly from cell images such as those in Figure 2. Unlike human expert reasoning, a CNN model does not extract features directly associated with interpretable morphological characteristics. However, through a structured network of convolutional filters, the images are processed to extract quantitative descriptors, which are used by a classifier to give an accurate cell class prediction. Learning is the key step in building the model. In this work, we use a database of images of lymphocytes obtained from the daily practice of a reference hospital and annotated by the consensus of three expert clinical pathologists to avoid variability. Furthermore, we define a rule to use the trained model in a clinical setting, where a patient’s smear is analyzed and the result is the prediction whether the patient belongs to a group associated with a better prognosis of the disease or not.

### 2.2. Model Selection

The first step was to select the appropriate structure for the classification system. We investigated three CNN structures pretrained with the ImageNet database [44]: EfficientNet B8 [45], RepVGG-B3g4 [46], and Xception71 [47]. Training and testing was performed in a server with a 12 GB Nvidia Titan XP Graphics Processing Unit (GPU). We performed a complete fine-tuning by training and testing the models using groups of patients and images whose details will be given later in Section 3.

We selected the CNN structure considering the accuracy of the tests (proportion of correctly classified images), training time, and implementation costs. For the model, the three CNN candidates showed high accuracy, above 90%. However, Xception71 increased the accuracy to 96.54%, while EfficientNet B8 and RepVGG-B3g4 showed almost the same accuracy of 92%. EfficientNet B8 was the network that took the longest to train, approximately 10 min per epoch. RepVGG-B3g4 and Xception71 had a reasonable training time for the problem addressed in this study, being 4 min per epoch.

Once the models were trained, we performed an additional test on the same server comparing the models deployed in operational mode. The system processed a total of 1491 cell images from the test set further detailed in Section 3.

For each network, the classification accuracy is shown in Table 1, along with the memory used by the GPU and the total execution time. To calculate this time, we used the “timeit” module in Phyton. We previously killed all background executions, closed non-essential programs, freed the memory, and checked the GPU temperature. Network daemons were also closed to minimize quantization error. Then, the set of 1491 test images were classified by the model in string form with 10,000 repetitions, taking the average execution time as the final result in Table 1.

From Table 1, Xception71 was selected for the model in the classification system of Figure 1. This architecture had the lowest computational cost on our GPU and the highest accuracy.

The following two subsections describe the model structure and the relevant details of the training process, respectively.

### 2.3. Model Structure

**Figure 3:**
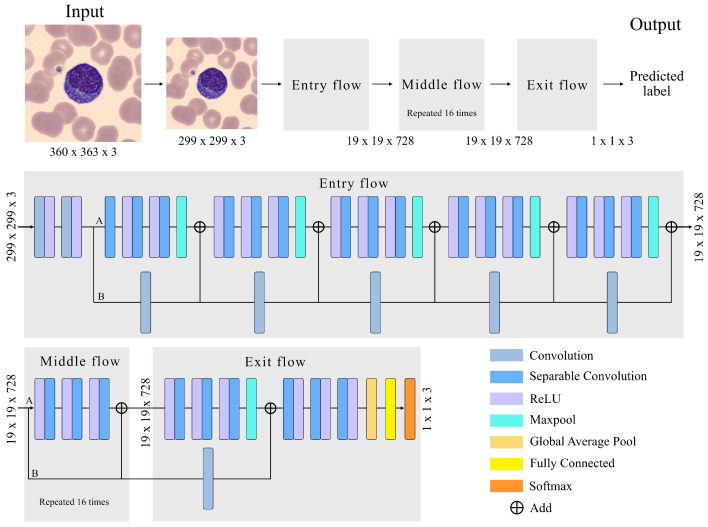
Model structure with 71 convolutions, 2 convolutions and 69 separable convolutions, a global average pool layer, a fully connected layer and a softmax output layer.

The adopted CNN has an Xception architecture, as shown in Figure 3. The Xception architecture is made up of three main parts: (1) Entry Flow, with six modules; (2) Middle flow, with sixteen modules; and (3) Exit flow, including two modules. The entire structure has a a total of 71 convolutional layers, trained to extract quantitative features that represent the images of the input cells. Complementing Figure 3, Table 2 and Table 3 provide the details of all modules and layers.

Before going through the Entry Flow, the size of our images is reduced from 360 × 363 × 3 (width, height, channel) to 299 × 299 × 3 because the implemented Xception71 architecture uses this size by default.

The images enter the first module, which is composed of two convolutional layers and two rectifier linear unit (ReLU) activation functions. The convolutional layer (Conv) is the most important unit for feature extraction. It is a structure that transforms an input volume into an output volume through a convolution operation.The convolution is the result of passing a kernel (filter) through the entire image in all its channels, obtaining the most relevant features in the learning process. This is repeated for the entire number of kernels. In our case, the convolutional layers of the first module have 32 and 62 filters, as can be seen in Table 2.

Parameter learning involves the gradient of the activation function. Sigmoid or hyperbolic tangent functions are monotone and differentiable, and were the default activation units used in neural networks for a long time. In both cases, the gradients vanish, which tends to slow down the learning process. This can make it difficult for multilayer networks to learn from training sets. In contrast, ReLU has a constant gradient and its use is trivial. Assigning an output value of zero for negative inputs is considered an additional benefit of ReLU, as it introduces sparsity into the network. This is a useful feature, as it can simplify the model and complete the learning process significantly faster than previous activation functions. Collectively, ReLU has become the practical default activation function in today’s deep neural networks.

In the second module of Entry Flow, a depthwise separable convolution (DSConv) is performed, represented in Figure 3 as the separation into two branches A and B. The DSConv was originally based on the Inception architecture [48], used to reduce the number of operations compared to a classical convolution, by performing convolutions in spatial dimensions (kernel) and in depth dimensions (channels). That is, it is composed of two types of convolutions, pointwise convolution (PConv) [49] and depthwise convolution (DConv) [49].

In branch A of module 2, three DConv are applied at the output of module 1. They are convolutions performed independently on each channel of the image, compressing its size in this process without affecting the number of channels. After performing these three convolutions, a max pooling layer is used to reduce the size of the feature map. This helps eliminate irrelevant details that are repeated in the input, reducing the sensitivity of the block to changes and distortions. The grouping is adjusted by two parameters: the size of the square part of the feature map whose values are grouped into a single number; and the stride, which is the number of steps along the height and width that the pool moves to cover the entire feature map. The size and stride values are in Table 2.

In branch B of module 2, a PConv of the output of module 1 is performed. It is a convolution of size 1 × 1 with a spatial depth equal to the input image. Its functionality is to pass 1 × 1 kernels along the image, obtaining at its output an image of original size and a spatial dimension increased by the number of kernels in the convolution. The two branches are joined with linear residual connections [50] represented in Figure 3 as “Add”. They are used in ResNet architectures. They allow jumping connections, avoiding gradient fading and higher error of training when more layers are added to the model. This connection is made in the entire model except for module 1 and module 24.

The structure of Module 2 is repeated in the subsequent modules 3–6 with an increasing number of kernel filters as detailed in Table 2.

The Middle Flow is made up of modules from 7 to 22, as detailed in Table 3. DConv is performed in each module so that the model learns a greater number of features. Each DConv is composed of three ReLU followed by separable convolutions. A PConv is not performed, as there is no need to increase the 728 channel dimensional space. The linear residual connection is maintained to prevent degradation (saturated training).

In the Exit Flow, a DSConv is performed followed by three DConvs increasing the number of filters to 2048. In module 24, we use a global average pool to determine the average of each feature map and link it to a fully connected layer. This layer has 2048 neurons. Each neuron performs an input-output operation of the form:

(1) $$z=f\left(b+\sum_{i=1}^{m}\omega_{i}x_{i}\right),$$

where $x_{i}$ are the input features, $w_{i}$ are the neuron weights, *m* is the number of input features, and *b* is a bias parameter.

The output layer has three nodes, which correspond to the final classification of the model in the recognition scheme of Figure 1. The softmax function is used to assign the class with the highest probability to the classification as follows:

(2) $$f_{sm}\left(y_{j}\right)=\frac{e^{y_{j}}}{\sum_{k=1}^{3}e^{y_{k}}},\;j=1,2,3.$$

### 2.4. Training Method

Training is an iterative process, where in each iteration the images from the training set are passed forward through the network. The results of the classification are compared to the ground truth labeled by clinical pathologists and used to calculate a loss function to quantify the error. Let us consider a set of *m* training images. In this work, the following categorical cross entropy loss was used:

(3) $$L=-\frac{1}{m}\sum_{i=1}^{m}\sum_{j=1}^{c}y_{j}^{\left(i\right)}\log\left({\hat{y}}_{j}^{\left(i\right)}\right),$$

where *c* is the number of classes, ${\hat{y}}_{j}^{\left(i\right)}$ is the probability of the predicted class, and $y_{j}^{\left(i\right)}$ is the label of the true class. This means that $y_{j}^{\left(i\right)}=1$ if the image sample *i* belongs to class *j*, and $y_{j}^{\left(i\right)}=0$ otherwise.

Since ${\hat{y}}_{j}^{\left(i\right)}$ depends on the weights and biases distributed in the network, the loss is a function of these parameters. For the sake of simplicity, all parameters are generically represented by θ. The training goal is to adjust θ to iteratively and gradually reduce the loss function towards its minimum using the gradient descent principle:

(4) $$\theta_{t}=\theta_{t-1}-\eta {\hat{g}}_{t},$$

where *t* represents the current iteration, $\hat{g}$ is an estimation of the gradient of *L* with respect to θ and η is the learning rate. Using the backpropagation approach, the gradient is calculated backwards through the network, first estimating the gradient with respect the parameters of the final layer and ending with the gradients corresponding to the first layer.

There are a variety of algorithms in the literature to optimize the learning process. In this study, we used the so-called Adam algorithm [51], which uses adaptive moment estimation, as is summarized below.

At any iteration *t*, the first step is the calculation of the gradient $g_{t}=\nabla_{\theta }L$, and then we calculate:

(5) $$v_{t}=\beta_{1}v_{t-1}+\left(1-\beta_{1}\right)g_{t},$$

(6) $$s_{t}=\beta_{2}s_{t-1}+\left(1-\beta_{2}\right)g_{t}^{2},$$

where $v_{t}$ and $s_{t}$ are the first and second moments of the gradient, respectively. These moments are initialized as v=s=0, so that the above recursive calculations are corrected as follows:

(7) $${\hat{v}}_{t}=\frac{v_{t}}{1-\beta_{1}^{t}},$$

(8) $${\hat{s}}_{t}=\frac{s_{t}}{1-\beta_{2}^{t}}.$$

The gradient is estimated as follows:

(9) $${\hat{g}}_{t}=\frac{{\hat{v}}_{t}}{\sqrt{{\hat{s}}_{t}}+\epsilon },$$

where ϵ is a very small parameter chosen for numerical stabilization, typically of the order $10^{-8}$.

Finally, the parameters are updated as:

(10) $$\theta_{t}=\theta_{t-1}-\eta {\hat{g}}_{t}.$$

## 3. System Training

This section is divided into two subsections. The first describes how the image database was compiled for the system development. The second presents the main results in the training/testing stage that ended with the classification system ready for implementation.

### 3.1. Cell Images

For the development of the classification system, we considered 18 patients with COVID-19 infection confirmed by a positive real-time reverse-transcription polymerase chain reaction (RT-PCR). They showed COVID-19 RL circulating in their blood. Peripheral blood smears were automatically prepared using the slide maker-stainer SP10i (Sysmex, Kobe, Japan) and stained with May Grünwald-Giemsa. The digital images of blood cells were acquired by CellaVision®DM96 (CellaVision, Lund, Sweden) (363 × 363 pixels) from smears collected during daily work at the Core Laboratory of the Hospital Clinic of Barcelona. These procedures are the same regardless of the technician working on them. Cell images were identified and annotated according to their morphological characteristics by the consensus of three experienced clinical pathologists.

A number of 187 COVID-19 RL images was obtained from the 18 patients. In addition, 4928 images of normal lymphocytes were collected from healthy controls and 2340 images of Classic RL were obtained from patients with other viral infections, which were used by the research group in previous works [22,24,35].

In summary, a total of 7455 digital cell images were available. The overall set was split into two subsets as shown in Table 4: 80% was randomly selected for training the models (5964 images), while the remaining 20% was saved for their testing (1491 images).

In general, training CNN models requires some balance of images from all classes. To compensate for the lower proportion of COVID-19 RL images, data was up-sampled by applying random transformations to the original images in the training set [28]:

- Image rotation from 0 to 120 degrees;
- Horizontal and vertical image flips;
- Maximum illumination in training images of 0.1;
- Maximum image zoom of 1.01.

Thus, we finally arranged a training dataset with 5000 images of normal lymphocytes, 5000 of Classic RL, and 5000 of COVID-19 RL (see Table 4).

### 3.2. Training Results

The system shown in Figure 1 was built using the CNN structure described in Table 2 and Table 3. The training was done using all the images up-sampled in Table 4: a fully balanced set with 5000 images for each class with its specific labels.

All the processes described in Section 2 were implemented in Python using the FastAI deep learning libraries. The Xception architecture was designed by its creator in TensorFlow and keras; however, FastAI’s Timm library adapted the architecture by using TensorFlow’s prebuilt weights and rebuilding the architecture under Pytorch. A 12 GB Nvidia Titan XP graphics processing unit was used.

In principle, the selection of the learning rate in the gradient descent scheme is crucial. High learning rates can have a regularization effect, preventing the network from overfitting and reducing accuracy. On the other hand, low learning rates can lead the model to a slow but more accurate decline in loss function. In this work, we used the cyclical learning rate policy [52]. This method practically eliminates the need to find the best value for the learning rate experimentally. It is inspired by the observation that increasing the learning rate could have a negative influence in the short term, while achieving a positive effect in the long term. The purpose is to set minimum and maximum limits and let the learning rate oscillate between these two values. It has been noted that training with cyclical learning rates rather than a fixed value achieves improved classification accuracy with fewer iterations [52].

**Figure 4:**
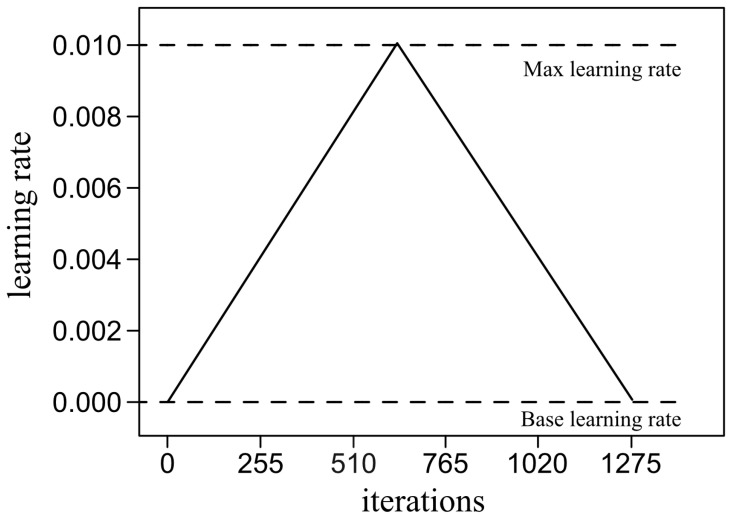
Learning cycle: number of iterations and bounding learning rates.

Figure 4 shows the triangular loop adopted in our training procedure. We split the (over-sampled) training set of Table 4 into two subsets: 85% to update the weights (12,750 images) and 15% (2250 images) to validate the updated models. For training, we consider an iterative scheme using a mini-batch of 10 randomly selected images without repositioning at each iteration. After 1275 iterations, all 12,750 images were used and one learning cycle was completed, which took 4 min. Once completed, the updated model ranked the 2250 images in the validation set to assess performance. This scheme was repeated for several cycles until the value of the loss function and the accuracy of the classification were acceptable.

The bounds of the learning rate η to define the learning cycle in Figure 4 were 0.001 and 0.01, respectively. Some previous learning trials were performed to check that, as the learning rate increased linearly from the lowest value, the accuracy increased until it began to decrease from 0.01. The remaining parameters of the Adam optimizer were $\beta_{1}=0.9$, $\beta_{1}=0.999$ and $\epsilon =10^{-8}$, which are typical values in many applications.

We trained the model with 36 learning cycles, observing that the loss of validation had a decreasing profile and the precision an increasing profile. We obtained 0.044 and 0.988, respectively, in the last cycle. With these results, we considered that the training of the entire classification system in Figure 1 was completed.

**Figure 5:**
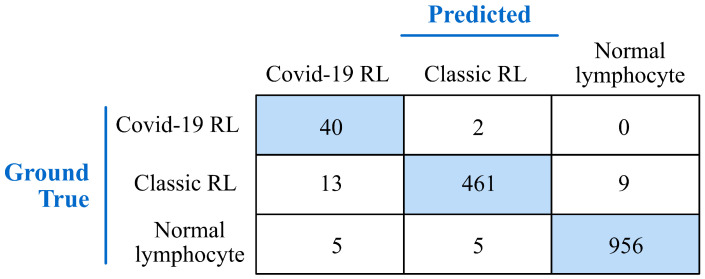
Confusion matrix for the classification of the test set. The cells are classified into normal lymphocytes, COVID-19 reactive lymphocytes, and classic reactive lymphocytes. The results are expressed in cell numbers.

The first evaluation of the system was carried out by a blind classification of the 1491 individual images in the test set (Table 4). Figure 5 shows the confusion matrix that summarizes the results of the model classifying the cells into Normal lymphocytes, COVID-19 RL or Classic RL. The rows give the values of ground true and the columns give the values predicted by the model. The main diagonal shows the true positive rate for each cell class.

From the confusion matrix, the following classifier performance metrics can be calculated:

$$\mathrm{Sensitivity}\;\left(TPR\right)=\frac{TP}{TP+FN}$$

$$\mathrm{Specificity}\;\left(TNR\right)=\frac{TN}{TN+FP}$$

$$\mathrm{Precision}\;\left(PPV\right)=\frac{TP}{TP+FP}$$

$$\mathrm{Overall}\;\mathrm{accuracy}\;=\frac{TP+TN}{TP+TN+FP+FN}$$

where *TP* and *TN* denote the number of predictions being true positive and true negative, respectively, and *FP* and *FN* the false positive and false negative predicted cases, respectively.

The above expressions correspond to a binary classification with a positive and a negative class. The classification in this work involved three cell types, with COVID-19 RL being the most relevant from a clinical point of view. Therefore, we calculated the performance metrics for COVID-19 RL as the positive class and the other two cell types together as the negative class. The following values were obtained:

Sensitivity=0.952

Specificity=0.988

Precision=0.689

Overallaccuracy=0.986

These metrics show that the model was very effective in separating COVID-19 RL, classic RL, and normal lymphocytes.

## 4. Experimental Assessment

The presence of COVID-19 RL cells circulating in the blood in patients with COVID-19 infection was shown to be an indicator of a better prognosis [10]. The purpose in this section was to assess whether the classification system presented in Figure 1 was capable of automatically recognizing the presence of a significant number of COVID-19 RL in blood smears from those patients. The first step was to arrange a cohort of patients and the corresponding cell images.

### 4.1. Patients for the Experimental Assessment

Clinical and laboratory findings from 185 patients infected with COVID-19 were compared. A number of 106 patients showed RL in blood (RL+ group) and these cells were absent in the remaining 79 patients (RL- group).

Blood samples were collected on admission to the Hospital Clinic of Barcelona, with several hematological and biochemical parameters being measured. Blood counts and biochemical parameters were analyzed in Advia2120i and Atellica, respectively (Siemens Healthcare Diagnostics SL). A Mann–Whitney U test and Fisher test were used for statistical analysis.

Dyspnea was more frequent in the RL- group (p=0.07). Hemoglobin, red blood cell, and lymphocyte (L) counts were higher in RL+ (p<0.001). In RL- patients, we found elevated values of neutrophils (N), N/L ratio, D-dimer, cardiac troponin I, procalcitonin, glomerular filtration rate, blood urea nitrogen, direct bilirubin, alkaline phosphatase, direct bilirubin, and lactic dehydrogenase (LDH) (p<0.001). All of these biomarkers have been related with a more severe COVID-19 infection [10].

Other parameters that increased significantly in the RL- group, and that were related to a worse evolution of the infection, were the platelet/leukocyte ratio (p<0.006), the number of monocytes (p=0.002), as well as the creatinine and gamma glutamyl transferase (GGT) values (p=0.005 and p=0.014, respectively).

In addition, RL- patients showed significantly decreased values of total protein and albumin (p<0.001). A high number of RL- patients received antibiotics (p<0.001), antifungals (p=0.013), and immunosuppressants (p=0.002). The number of days of hospitalization and the period between the onset of symptoms and discharge was greater for RL- patients (p<0.001). In this group, patients who required admission to the intensive care unit or required mechanical ventilation and mortality were higher (p<0.001).

It was found that RL detection in the blood smear is related to a better prognosis of the COVID-19 infection, suggesting an abundant production of virus-specific T cells, thus explaining the better outcome of patients showing these cells in blood.

### 4.2. Images for the Experimental Assessment

Among the group described in the previous subsection, we obtained images from 92 patients with a single smear available for each individual. None of the patients were used in any of the steps involved in the model development. All the digital images were acquired as described in Section 3.1 for the system training. The following groups were defined:

- COVID-19 RL-positive group: This is the group associated with a better disease prognosis. It included 58 patients with COVID-19 infection confirmed by positive real-time RT-PCR, whose smears contained both COVID-19 RL and normal lymphocytes. In addition, Classic RL were also present in the smears of 27 patients, with a total of 70.
- COVID-19 RL-negative group: This group includes 34 patients with COVID-19 infection confirmed by positive real-time RT-PCR, whose smears did not contain COVID-19 RL. This is the group associated with the worst disease prognosis. Most of the patients presented exclusively normal lymphocytes, but in 6 of them, between 1 and 4 Classic RL were counted, with a total of 12.

Table 5 shows the cell image distribution in each group of patients.

### 4.3. Experimental Results

In this study, the entire blood smear was the test unit. This means that the input was a set of lymphoid cell images from an individual smear selected by the clinical pathologist, trying to emulate the way they interpret results in clinical laboratories. The result was the classification of the smear into one of the groups under study, which gave a prediction about the prognosis of the patient.

Note that the two groups in Table 5 have in common that both include patients diagnosed with COVID-19 infection. The main difference is that patients in the positive group have COVID-19 RL. In the negative group, patients do not have COVID-19 RL, as their immune systems have not produced virus-specific T cells for their defense. All smears include normal lymphocytes, as usual in blood samples.

Since the main goal of the classification system was to identify the presence of COVID-19 RL in blood smears from COVID-19-infected patients, the positive group became the primary target. For the system to identify a smear as belonging to the positive group, it must recognize a minimum number of COVID-19 RL cells.

**Figure 6:**
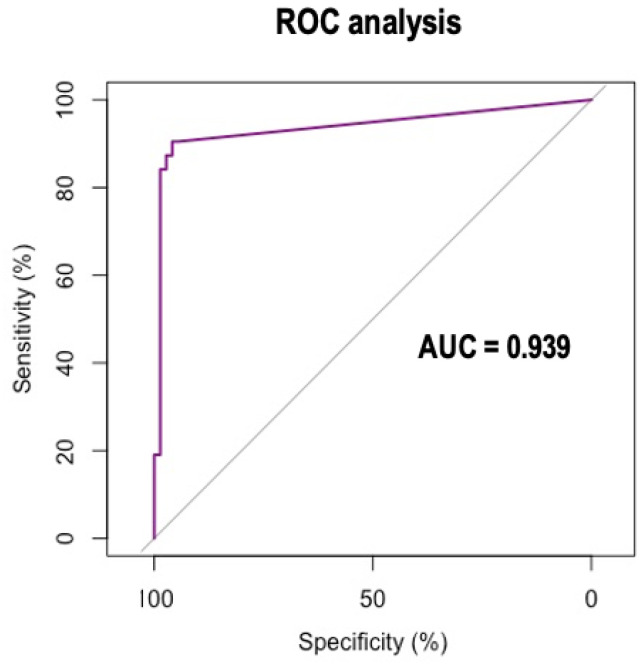
ROC analysis to obtain the best threshold (2%) to predict whether a smear belongs to the COVID-19 RL-positive group or not.

To do this, we carried out an experiment in which all the smears described in Table 5 were analyzed by the system in Figure 1 considering a threshold value for the identification of the positive group. By varying the threshold and comparing the classification with the ground truth, a Receptor Operational Characteristics (ROC) analysis was carried out using the statistical software R. Figure 6 shows the ROC curve obtained. It was found that 2% of the cell images correctly classified as COVID-19 RL was the best threshold to predict that the smear belongs to the COVID-19 RL-positive group. The value of the area under the curve was 0.939, which supports that the threshold obtained was adequate.

Once this threshold was determined, the classification system was finally evaluated by blind classification of all the smears in Table 5. For a given smear, all its cell images were classified and a prediction was made about the group to which it belonged according to the following rules:

- COVID-19 RL-positive group if the number of COVID-19 RL cells was above the threshold;
- COVID-19 RL-negative group otherwise.

**Figure 7:**
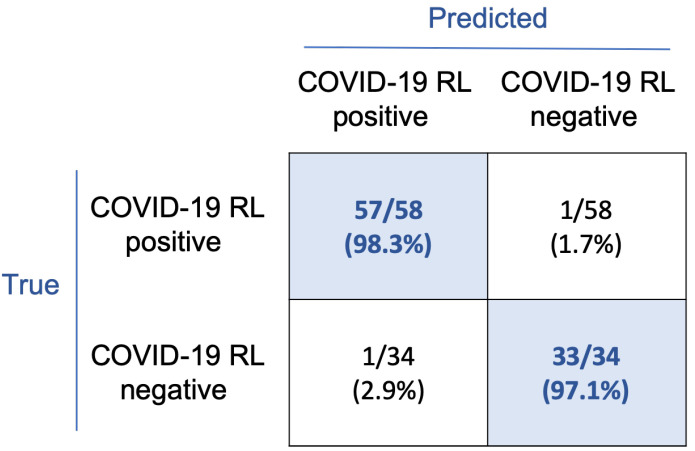
Confusion matrix for the classification of the 92 smears from the infected patients under study. Target groups are COVID-19 RL-positive and COVID-19 RL-negative. Results are expressed in absolute values and in percentages.

The confusion matrix in Figure 7 shows the classification results. With this rule, 57/58 smears corresponding to the COVID-19 RL-positive group and 33/34 to the COVID-19 RL-negative group were correctly classified. Considering the COVID-19 RL-positive group as the main clinical target, sensitivity and precision both are 98.3%, specificity is 97.1%, and overall accuracy is 97.8%.

## 5. Discussion

The work presented in this paper was motivated by: (1) the observation of COVID-19 RL circulating in peripheral blood in some of the patients infected with COVID-19; and (2) the hypothesis that deep learning models could aid in their accurate, objective, and rapid automatic recognition.

The presence of COVID-19 RL in peripheral blood has clinical relevance, since it was found that they are related to a better prognosis of the disease and a better evolution of the patients. In fact, a comparative study [10] between two groups of COVID-19-infected patients, with and without COVID-19 RL circulating in blood, concluded that: (1) the number of days in hospital was significantly lower for patients with COVID-19 RL in blood, as well less time between onset of symptoms and discharge; and (2) the number of patients who required mechanical ventilation or died due to severe acute respiratory problems were lower. Overall, patients carrying COVID-19 RL in their blood had a more effective immune response against virus infection. Therefore, the early recognition of these reactive cells based on the morphological analysis of the blood smear can help in the detection of critical illness stage and may support a provisional clinical prognosis of COVID-19 infection [5].

Cell morphology has proven to be crucial for the initial diagnosis of serious diseases, such as different types of leukemia, lymphoma, and myelodysplastic syndromes, among others. Machine learning and CNN models have been increasingly proposed as tools to help clinical pathologists achieve early diagnostic orientations, as summarized in Section 1.

The present work addressed the development of a new system for the automatic recognition of COVID-19 RL cells with the final focus on the prognosis of the disease of infected patients. The main challenge was faced with respect to morphological differentiation, given the similarity between the two classes of reactive cells involved: COVID-19-RL and classic RL, also found in other viral infections [6,7,8,9].

Our strategy was to train a three-class convolutional neural network with our own database of cell images. The architecture was Xception71, one of the recent successful models available in the literature, selected in comparison with other similar frameworks. It is efficient for our problem in terms of accuracy, memory, and execution time.

An important aspect to discuss in this work is the quantity and quality of the images. As seen from the dataset in Table 4, the number of available images of COVID-19 RL was 145 for training and 42 for testing. It is common that, in medical applications, samples from patients are scarce. In the case of COVID-19, being a new disease, this problem is particularly understandable. To compensate for the unbalanced dataset, in this work, data augmentation was carried out by using random transformations to the original images for training. In a previous work [28], it was shown that this type of balancing was effective to stabilize the training loss function with a high accuracy compared to the use of unbalanced training sets. Besides quantity, images should have good quality and be properly annotated to allow the observation of morphological characteristics, useful in the daily clinical practice and also to develop robust classification models avoiding overfitting. In general, medical data are difficult to annotate. In our interdisciplinary research group, we have experienced pathologists able to guarantee a manual labeling that is also confirmed through other complementary tests (ground truth). On the other hand, images were all stained with the same standard May Grünwald–Giemsa technique used in the clinical practice.

Regarding the practical use of the classification system for prognostic purposes, we designed a strategy focused on patients diagnosed with COVID-19 confirmed by real-time RT-PCR. The objective was to classify a complete set of cells from a patient’s smear into two possible groups: one with a better prognosis and one with a worse prognosis. It was found that an accurate classification was obtained after determining a threshold of 2% for the COVID-19 RL cells recognized by the system in the smear.

Table 5 shows that the 58 study patients belonging to the positive group (good prognosis) have a total of 132 COVID-19 RL cells, 70 classic RL, and 1604 normal lymphocytes. This means that, on average, a smear from a patient in the positive group has between 2 and 3 COVID-19 RL cells, between 0 and 1 classic RL, and between 27 and 28 normal lymphocytes. The 2% threshold means that, on average, the system correctly predicts a good prognosis when it identifies at least one COVID-19 RL cell in the smear. On the other hand, Table 5 shows that the 34 patients in the negative group have a total of 420 normal lymphocytes (about 8 cells on average), while they do not have COVID-19 RL. The threshold is so stringent that the system would misidentify the smear as belonging to the positive group simply by identifying a single true normal cell as a COVID-19 RL. In this regard, it should be noted that the classification system achieved an accuracy of 956/966 = 99% in the separation of single cells among normal lymphocytes, COVID-19 RL, and classic RL (see Figure 5).

The threshold was very effective in classifying the smears, as shown in Figure 7. The sensitivity (98.3%) and specificity (97.1%) values obtained in the experimental evaluation of patient smears are high enough to support the possible application of the proposed system in a clinical setting of this type. It could be a tool to help in the early detection of COVID-19 reactive lymphocytes in peripheral blood and, consequently, to confirm the better prognosis of patients compared to those without these cells.

The system is inexpensive from a computational point of view and could easily be implemented to operate in real time as a rapid tool in the initial stage of a patient’s diagnosis. The selected images corresponding to the lymphocytes of the patient under study can be sent by the clinical pathologist to the system implemented for prognosis prediction.

The work has limitations related to the number of patients and images involved. Not all COVID-19-infected patients have these reactive lymphocytes and the number is low. In this work, we implemented simple techniques for data augmenting. Although they have been satisfactory for the present study, more sophisticated techniques may be used in future works [53].

The model proposed in this work was trained using a database of images acquired in a specific laboratory using a standardized acquisition (CellaVision) and staining procedure (May-Grünwald-Giemsa). Images were annotated according to their morphological characteristics by the consensus of three experienced pathologists to avoid variability Therefore, the algorithm is ready for any new image set prepared with the same standard regardless of the laboratory. However, there may be variability in staining results between laboratories depending on the ratio and concentration of the chemicals, the duration of their contact with the smear, and other similar factors. This variability can lead to inconsistency among pathologists in their visual inspection and, likewise, can affect the performance of automatic recognition systems [54]. More work is being done to compensate for this variability using adversarial networks.

In addition to visual morphology, various hematological and biochemical variables are obtained from blood samples, which may be related to the prognosis and the favorable clinical course of the illness. All these types of prognostic results are not disclosed to the patient; they are used exclusively by clinicians. The combination of these variables with features obtained from the images could be explored to develop more complete prediction models with additional work.

## 6. Conclusions

To the authors’ knowledge, this article presents the first CNN-based computational model in the literature for the morphological detection of COVID-19 reactive lymphocytes and its implications in an early prognostic prediction of COVID-19 infection. The model has been successfully implemented and tested in a group of patients.

The model does not require costly computations and could be potentially integrated in clinical practice to assist clinical pathologists in a more objective smear review. In this study, all the patients came from the same hospital. It would be interesting to extend the study and include patients and images from other centers to broaden training and generalize the scope of the models to broader applications. This could help bring our system closer to practical application in a clinical laboratory.

## Figures and Tables

**Table 1:** Monitoring the implementation of the system for three CNN candidates.

	Accuracy (%)	Memory (MB)	Execution Time (s)
EfficientNet B8	92.3	929	76.37
RepVGG-B3g4	92	921	26.92
Xception71	96.54	797	32.93

**Table 2:** Structure of the Entry Flow of the CNN model. All separable convolutional filters have a size of 3×3. All maxpool layers use the size F=3.

Input: 299×299×3				
**Entry Flow**				
**Layers**		**Number N of Filters**	**Size**	**Number of Module**
	Conv	32		
	ReLU			
	Conv	64	3 × 3	1
	ReLU			
	Separable Conv	128		
	ReLU			
Conv 1 × 1	Separable Conv	128	3 × 3	2
stride = 2 × 2	ReLU			
	Separable Conv	128		
	Max Pooling			
	ReLU			
	Separable Conv	256		
Conv 1 × 1	ReLU			
stride = 1 × 1	Separable Conv	256	3 × 3	3
	ReLU			
	Separable Conv	256		
	Max Pooling			
	ReLU			
	Separable Conv	256		
Conv 1 × 1	ReLU			
stride = 2 × 2	Separable Conv	256	3 × 3	4
	ReLU			
	Separable Conv	256		
	Max Pooling			
	ReLU			
	Separable Conv	728		
Conv 1 × 1	ReLU			
stride = 1 × 1	Separable Conv	728	3 × 3	5
	ReLU			
	Separable Conv	728		
	Max Pooling			
	ReLU			
	Separable Conv	728		
Conv 1 × 1	ReLU			
stride = 2 × 2	Separable Conv	728	3 × 3	6
	ReLU			
	Separable Conv	728		
	Max Pooling			
Output: 19×19 × 728 feature maps				

**Table 3:** Structure of the Middle and Exit Flow of the CNN model. All separable convolutional filters have a size of 3×3. All maxpool layers use the size F=3.

Input: 19×19 × 728 Feature Maps				
**Middle Flow**				
**Layers**		**Number N of Filters**	**Size**	**Number of Module**
	ReLU		3 × 3	7–22
	Separable Conv	728		
	ReLU			
	Separable Conv	728		
	ReLU			
	Separable Conv	728		
	Max Pooling			
Output: 19×19 × 728 feature maps				
Exit Flow				
Layers		Number N of Filters	Size	Number of Module
	ReLU			
	Separable Conv	728		
Conv 1 × 1	ReLU			
stride= 2 × 2	Separable Conv	1024	3 × 3	23
	ReLU			
	Separable Conv	1024		
	Max Pooling			
	Separable Conv	1536		
	ReLU			
	Separable Conv	1536	3 × 3	
	ReLU			
	Separable Conv	2048		
	ReLU			24
Global Average Pooling				
2048 dimensional vector				
Fully Connected Layer				
1000 fully connected nodes				
SoftMax function				
Output: three classes prediction				

**Table 4:** Image datasets used for training and testing.

	Training (Initial)	Testing	Training (Up-Sampled)
Normal lymphocytes	3962	966	5000
Classic RL	1857	483	5000
COVID-19 RL	145	42	5000
TOTAL	5964	1491	15,000

**Table 5:** Image dataset used for the experimental assessment. Number of images of the three classes of cells under study: COVID-19 RL, classic RL, and normal lymphocytes.

Patients	Group	COVID-19	Classic	Normal	
		RL	RL	Lymphocytes	Total
58	COVID-19 RL-positive	132	70	1604	1806
34	COVID-19 RL-negative	-	12	420	432
92	TOTAL	132	82	2024	2238

## Data Availability

Data relevant to the study are included in the article. Image sharing not applicable.
